# Case report: Resolution of oligo-anuric acute kidney injury with furosemide administration in a cat following lily toxicity

**DOI:** 10.3389/fvets.2023.1195743

**Published:** 2023-07-05

**Authors:** An To, Claudia Davila, Sarah Stroope, Rebecca Walton

**Affiliations:** ^1^Department of Emergency and Critical Care, Veterinary Centers of America (VCA) West Los Angeles Animal Hospital, Los Angeles, CA, United States; ^2^Department of Emergency and Critical Care, Iowa State University, Ames, IA, United States

**Keywords:** anuria, acute kidney injury, lily (*Lilium* and *Hemerocallis* spp.), furosemide, toxicity, oligo-anuria

## Abstract

**Objective:**

To describe the successful outcome of a case of oligo-anuric acute kidney injury in a cat secondary to lily ingestion.

**Case summary:**

A 12-week-old intact male domestic short-hair cat weighing 1.64 kg (3.6 lb) presented with a 12-h duration of vomiting and lethargy after exposure to lilies of the genera *Lilium* species 24 h prior to presentation. Severe azotemia (Creatinine 5.8 mg/dL, BUN > 100 mg/dL) and hyperkalemia (9.36 mmol/L) were noted on the day of presentation. Treatment of hyperkalemia was instituted with calcium gluconate, lactated ringers solution, dextrose, regular short-acting insulin, albuterol, and sodium bicarbonate, Oliguria to anuria was highly suspected based on a lack of urine production 21 h after hospitalization with intravenous fluid administration and a static bladder size. The cat was administered 4 mg/kg of furosemide, and urinated at 6 h following administration and continued to produce over 6 ml/kg/h of urine in the next 24 h. Two days following furosemide administration, the cat's hyperkalemia and azotemia resolved. The cat was discharged after 4 days of hospitalization, and a recheck revealed no persistent azotemia or hyperkalemia.

**Unique information:**

Anuric acute kidney injury secondary to lily toxicity is associated with a poor prognosis, and the only treatment modality previously described is hemodialysis. The cat in this report was successfully managed with medical intervention and furosemide administration, with complete resolution of the acute kidney injury.

## Introduction

Lilies, including the genera *Lilium* and *Hemerocallis*, are known to cause acute kidney injury (AKI) in cats (1). Clinical findings associated with lily toxicity consist of gastrointestinal signs, lethargy, polyuria, polydipsia, azotemia, glucosuria, proteinuria, and in severe cases, oliguria or anuria (2). When diagnosed early, lily toxicity carries an excellent prognosis (3). Survival rates up to 100% are observed when gastrointestinal decontamination and supportive fluid therapy are initiated within 48 h of exposure (3). However, the prognosis is poor if cats develop anuric renal failure (1). The exact mechanism of action of lily toxicity is unknown; however, toxicity results in severe renal tubular damage and cellular death with gross lesions, including renal congestion, peri-renal edema, and renal tubular necrosis (1). Polyuric AKI occurs 12–30 h following ingestion, and anuric AKI may develop between 24 and 48 h (1). Historically, renal replacement therapy, including peritoneal dialysis and hemodialysis, have been the only effective forms of treatment for anuria secondary to lily toxicity, with medical management not proven effective (4). Renal replacement therapies are not readily available in veterinary medicine and are often cost-prohibitive. Medical management of anuric AKI includes the use of diuretics, most commonly furosemide, and historically mannitol (5). Furosemide is transported to the luminal membrane of the renal proximal tubule by renal organic anion transporters and inhibits sodium-chloride-potassium cotransporters of the ascending limb of the loop of Henle (6). Furosemide causes increased urine output but has not been shown to increase glomerular filtration rate or improve outcomes (7). Mannitol has also been shown to increase urine output but has potential adverse effects, including fluid overload and the development of osmotic nephrosis, and is not currently recommended (5, 8). To the authors' knowledge, there are no reported cases of spontaneous conversion from oligo-anuria to polyuria or successful management of anuric AKI in a cat with lily toxicity without renal replacement therapy. This case describes the successful outcome and conversion of oligo-anuric AKI to polyuric AKI in a cat with lily toxicity.

## Case summary

A 12-week-old intact male domestic short-hair cat weighing 1.64 kg (3.6 lb) presented to the Emergency Department of a large private practice hospital for the evaluation of acute onset of vomiting and lethargy over a 12-h duration. The cat was an indoor-only cat with a history of flea anemia months prior; however, there was no other history or current medications at the time of presentation. The attending veterinarian identified that the cat was exposed to a bouquet of flowers containing Easter lilies of the genera *Lilium* species 24 h prior to presentation. The cat's appetite had been normal 24 h prior to presentation. The cat was initially evaluated by the primary care provider, and a chemistry profile performed 12 h after the onset of vomiting revealed severe azotemia with a creatinine of 6.0 mg/dL; 530 μmol/L (reference range 0.6–1.6 g/dL; 53–141 μmol/L), blood urea nitrogen (BUN) 119 mg/dL; 42.5 μmol/L (reference range 16–33 mg/dL; 5.7–11.7 μmol/L), phosphorus > 16.1 mg/dL; 5.1 μmol/L (reference range 4.5–10.4 mg/dL; 1.4–3.4 μmol/L), Na 146 mmol/L (reference range 150–165 mmol/L), K 9.6 mmol/L (reference range 3.7–5.9 mmol/L), and glucose 217 mg/dL; 12 mmol/L (reference range 77–153 mg/dL; 4.3–8.5 mmol/L) (Table 1). No treatment was instituted prior to referral.

**Table 1:** Biochemical values and body weight during hospitalization and recheck.

	**Day 0 11:25**	**Day 0 12:00**	**Day 0 16:00**	**Day 0 21:30**	**Day 1 6:00**	**Day 1 17:30**	**Day 2**	**Day 3**	**Day 4**	**Day 6**
BUN (mg/dL)	119	>100	>100	>100	>100	>100	54	34	21	23
Creatinine (mg/dL)	6.0	5.8	3.5	5.8	3.9	4.4	1.7	1	0.8	0.9
Na (mmol/L)	146	138.4	137.3	138.4	135.8	143.9	153.3	153.5	154.4	150
K (mmol/L)	9.6	9.6	8.41	9.36	9.93	7.59	5.12	5.12	4.74	4.93
Glucose (mg/dL)	217	146	89	146		90	93	93	115	81
Body weight (Kg)	1.64	1.64	1.74	1.75	1.76	1.75	1.75	1.88	2	1.97

The patient was normothermic at 37.6 C; 99.7 F, bradycardic at 140 beats per minute, and had a respiratory rate of 50 breaths per minute on presentation. Mucous membranes were tacky and light pink, with a capillary refill time of < 2 s. Physical examination at the time of presentation (day 0) was consistent with 5% dehydration based on tacky mucous membranes and a mild decrease in skin turgor. The bradycardia was attributed to hyperkalemia, with no evidence of hypovolemia noted. The patient was dull with no noted neurologic deficits and bradycardic with no appreciated murmur. A small but palpable bladder was noted at the time of presentation. The patient's weight was 1.64 kg upon admission. A diagnostic electrocardiogram revealed sinus bradycardia with wide QRS complexes. On venous blood gas, a hyponatremia at 138.4 mmol/L (reference range 146.2–156.2 mmol/L), hyperkalemia 9.36 mmol/L (reference range 3.42–4.71 mmol/L), ionized hypocalcemia 1.07 mmol/L (reference range 1.16–1.35 mmol/L), blood urea nitrogen (BUN) >100 mg/dL (reference range 8.0–30.0 mg/dL), and creatinine of 5.8 mg/dL; 512.8 mmol/L (reference range 0.6–1.6 g/dL; 53–141 μmol/L) were noted. Urine specific gravity was 1.018, and no evidence of calcium oxalate monohydrate crystals was noted on direct urine sediment examination. Initial therapeutics consisted of calcium gluconate^1^ 1 mL/kg diluted 1:4 with 0.9% saline IV over 20 min, 10 mL/kg lactated ringer's solution^2^ IV bolus, 1 mL/kg of 50% dextrose^3^ diluted 1:4 with 0.9% saline IV over 5 min, regular, short-acting insulin 1 unit IM, maropitant^4^ 1 mg/kg IV, 1 puff of albuterol^5^ to treat patient's hyperkalemia, and 1 mL/kg of sodium bicarbonate 8.4%^6^ diluted 1:5 with D5W IV over 10 min. The cat was started on a lactated ringer's solution (see text footnote 2) at a rate of 9 mL/kg/h with 5% dextrose, due to insulin administration, for 3 h based on 70 mL/kg/day maintenance in addition to 5% dehydration aimed to be replaced over 6 h followed by 73 mL/kg/day with 5% dextrose (see text footnote 3). Additionally, ampicillin/sulbactam^7^ (30 mg/kg IV q8h) was added to the therapeutic plan. The cat was monitored overnight, including telemetry monitoring and body weight measurement every 4 h. Venous blood gas performed 4 h post-presentation revealed hyponatremia at 137.3 mmol/L (reference range 146.2–156.2 mmol/L), improved hyperkalemia at 8.41 mmol/L (reference range 3.42–4.71 mmol/L), ionized hypocalcemia 1.1 mmol/L (reference range 1.16–1.35 mmol/L), and improved azotemia with a creatinine of 3.5 mg/dL; 344 μmol/L (reference range 0.6–1.6 g/dL; 53–141 μmol/L). Abdominal ultrasound performed revealed bilaterally hyperechoic renal cortexes with normal renal architecture. On venous blood gas performed 10 h following initial evaluation, hyponatremia at 133.0 mmol/L (reference range 146.2–156.2 mmol/L), hyperkalemia at 9.49 mmol/L (reference range 3.42–4.71 mmol/L), and ionized hypocalcemia 1.07 mmol/L (reference range 1.16–1.35 mmol/L) were noted with a creatinine of 5.8 mg/dL; 513 μmol/L (reference range 0.6–1.6 g/dL; 53–141 μmol/L). The patient's weight was 1.74 kg at that time. An additional dose of regular, short-acting insulin^8^ at 0.25 U/kg was given intravenously along with 2 puffs of albuterol (see text footnote 5). Hospitalization continued, and no urination was noted during the first 15 h of hospitalization; at this time, the bladder size was subjectively measured via abdominal ultrasound, revealing a size of 4.2 × 4.5 cm.

**Figure 1:**
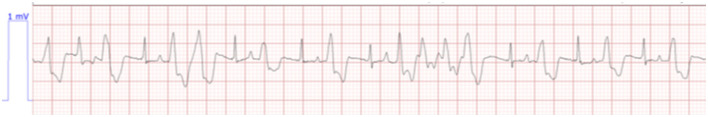
Bradycardia with intermittent ventricular tachycardia. Lead II, HR 127, paper, speed 25.0 mm/s.

**Figure 2:**
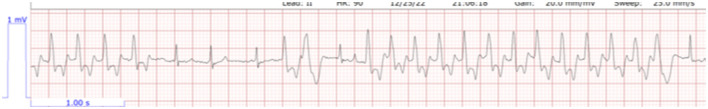
Bradycardia with more frequent runs of ventricular tachycardia. Lead II, HR 90, paper speed 25.0 mm/s.

On day 1 of hospitalization, 18 h post-presentation, the cat continued to have a dull mentation with a significant appreciation of abdominal pain. When offered food, the cat would eat readily. Telemetry at this time revealed bradycardia with intermittent ventricular tachycardia (Figure 1). The cat's weight was static at 1.76 kg. On venous blood gas, a hyponatremia at 135.8 mmol/L (reference range 146.2–156.2 mmol/L), hyperkalemia 9.93 mmol/L (reference range 3.42–4.71 mmol/L), and ionized hypocalcemia 1.07 mmol/L (reference range 1.16–1.35 mmol/L) were noted with a creatinine of 3.9 mg/dL; 345 μmol/L (reference range 0.6–1.6 g/dL; 53–141 mmol/L). Treatment at this time consisted of lactated ringer's solution (see text footnote 2) at 75 mL/kg/day based on a maintenance rate for a young animal, with 5% dextrose (see text footnote 3), maropitant (see text footnote 4) 1 mg/kg IV q24h, pantoprazole^9^ 1 mg/kg IV q12h, and albuterol (see text footnote 5) 2 puffs every 8 h. Twenty-one hours post-presentation, the cat began having longer and more frequent runs of ventricular tachycardia (Figure 2), and treatment with terbutaline^10^ at 0.01 mg/kg IM every 8 h was initiated as continued management for hyperkalemia. At this time, bladder measurement was static at 4.2 × 4.5 cm. Due to worsening cardiac status, lack of urine production, and no change in bladder size over the initial 21 h of hospitalization, a dose of furosemide (see text footnote 7) at 4 mg/kg was administered intravenously. Six hours following the furosemide administration, the cat urinated approximately 4 ml/kg/h. Venous blood gas performed 30 min following urination (6.5 h following furosemide administration and 28 h post initial evaluation) revealed sodium of 143.9 mmol/L (reference range 146.2–156.2 mmol/L), improved hyperkalemia at 7.59 mmol/L (reference range 3.42–4.71 mmol/L), and ionized hypocalcemia 1.07 mmol/L (reference range 1.16–1.35 mmol/L) with a creatinine of 4.4 mg/dL; 389 μmol/L (reference range 0.6–1.6 g/dL; 53–141 μmol/L). Ongoing treatment included isotonic crystalloid (see text footnote 2) at 75 mL/kg/day, ampicillin/sulbactam^11^ 30 mg/kg IV q8h, and maropitant (see text footnote 4) 1 mg/kg IV q24h.

On day 2 of hospitalization, the cat urinated over 8 times, totaling over 6 ml/kg/h. The weight ranged from 1.74 to 1.96 kg, and the patient was noted to have a normal sinus rhythm and a good appetite. Ampicillin/sulbactam (see text footnote 7) was discontinued on day 2 of hospitalization due to negative urine culture results. The cat continued to eat well, and during the morning physical examination, the cat was bright and comfortable on abdominal palpation. Venous blood gas revealed a sodium of 153.5 mmol/L (reference range 146.2–156.2 mmol/L), improved hyperkalemia at 5.12 mmol/L (reference range 3.42–4.71 mmol/L), ionized calcium of 1.39 mmol/L (reference range 1.16–1.35 mmol/L), and improved azotemia with a creatinine of 1.7 mg/dL; 150 μmol/L (reference range 0.6–1.6 g/dL; 53–141 μmol/L). Treatments on day 2 of hospitalization included isotonic crystalloids (see text footnote 2) at 300 mL/kg/day to match urine output and maropitant (see text footnote 4) 1 mg/kg IV q24h. The patient remained clinically hydrated with unremarkable vital signs and physical exam findings.

On day 3 of hospitalization, venous blood gas revealed potassium of 5.12 mmol/L (reference range 3.42–4.71 mmol/L) and creatinine of 1.0 mg/dL; 88 μmol/L (reference range 0.6–1.6 g/dL; 53–141 μmol/L). Intravenous fluids were tapered by a 25% reduction every 6 h throughout days 3 and 4 of hospitalization, and the patient was discharged on day 4 with a creatinine of 0.8 mg/dL; 70.4 mmol/L (reference range 0.6-1.6 g/dL; 53–141 μmol/L) and potassium of 4.74 mmol/L (reference range 3.42–4.71 mmol/L). The patient was evaluated 2 days following discharge (day 6). The patient was clinically normal at home, eating and drinking, with no concerns noted. Bloodwork during recheck revealed a creatinine of 0.9 mg/dL; 79.5 mmol/L (reference range 0.6–1.6 g/dL; 53–141 μmol/L), and potassium of 4.93 mmol/L (reference range 3.42–4.71 mmol/L). During the time of writing, 3 months following ingestion, the patient is asymptomatic at home and with no changes or concerns since recheck.

## Discussion

Lily toxicity results in nephrotoxic tubular necrosis with selective damage to the renal tubular epithelium (1, 2, 9). Clinical signs are acute, starting with gastrointestinal signs within 1–3 h post-exposure, polyuria within 12–30 h, and anuria can occur starting from 24 to 48 h (1). The mainstay of therapy for lily toxicity is the administration of intravenous fluids to address dehydration and maintain euvolemia to support renal perfusion. Once anuria develops, hemodialysis is the only treatment modality that has been used in cats with lily toxicity. However, the prognosis is still poor, with survival of ~18% in cats treated with hemodialysis (5, 10). In addition to the poor prognosis associated with hemodialysis in lily toxicity in cats, hemodialysis may be associated with systemic complications, including hypotension, as the volume of pediatric blood lines and dialyzers approximate the total blood volume of cats resulting in a significant financial burden (11). The cat in this case report would have had an increased risk of complications due to its size, and hemodialysis is not described in cats < 2.4 kg (4, 11, 12).

Conversion attempts of oliguric or anuric AKI to polyuria include the use of the diuretic furosemide, a loop diuretic (7). Furosemide inhibits chloride transport resulting in natriuresis, kaliuresis, calciuresis, and increased urine flow (6, 13). It may additionally provide protection from ischemic damage by reducing sodium reabsorption or decreasing renal oxygen consumption through the blockade of the Na+-K+-ATPase dependent pump (6, 13). The combination of natriuresis, kaliuresis, and calciuresis makes furosemide useful in reducing hyperkalemia and fluid overload (14). Response to furosemide is also an indicator of functional tubular cells and can be used as a predictor of the severity of AKI (13). Human studies demonstrate that a lack of response to furosemide is associated with an increased risk of AKI progression and an increase in the need for renal replacement therapy (13). The efficiency of furosemide is determined by its ability to reach the tubular lumen, and AKI may require higher doses to achieve therapeutic levels due to a combination of reduced tubular secretion of furosemide and a blunted response of the Na-K-Cl_2_ cotransporters (6, 14). Due to potential side effects, such as aciduria, ototoxicity, and the precipitation of vasoconstriction, furosemide administration in people was previously controversial (14). Previous human studies have not demonstrated a reduction in risk of requiring renal replacement therapy or mortality, and furosemide administration can decrease water reabsorption and increase urine output without improvement of creatinine and renal function (14, 15). More recent studies, however, demonstrate improved short-term survival, recovery of renal function in AKI, and improvement of azotemia in oliguric patients who received furosemide (13, 16–19). More recently, the furosemide stress test (FST), which evaluates urine production in response to an IV bolus of furosemide, predicts the progression of AKI and decreases the need for renal replacement therapy. In the early stages, FST can predict progressive AKI better than biochemical markers (16, 20). There are few studies in veterinary medicine looking at the effect of furosemide administration on AKI. In a retrospective study of dogs with leptospirosis, two of 14 dogs with oliguria became polyuric after furosemide administration (21). Another study showed that the combination of furosemide and dopamine had improved urinary output and diuresis with no changes in renal blood flow in healthy cats (19, 21). Despite increasing evidence to support the role of furosemide in people with AKI, there are no studies evaluating the effect of furosemide on urine output in cats with AKI (19, 21).

The cat in this case report was deemed oligo-anuric based on a lack of urine production 21 h after hospitalization with IV fluid administration, after correcting for dehydration, and a static bladder size of 4.2 × 4.5 cm. Six hours following the administration of 4 mg/kg of furosemide IV, the cat was noted to urinate and continued to urinate subsequently at ~6.8 mL/kg/h over the first 24 h, in comparison to a normal urine output of 1–2 mL/kg/h. The patient's azotemia resolved within 6 days of hospitalization and 5 days after furosemide administration. Although the timing of furosemide administration coincided with urine output production, it is also possible that the patient converted from oligo-anuria to non-anuria without intervention. Additionally, it was suspected that the cat in this case report was oligo-anuric based on lack of urination and a static bladder size on ultrasound assessment. However, urine output was not measured via urinary catheterization but rather by weighing urinary pads.

This is the first case to describe the successful outcome of oligo-anuric AKI in a cat secondary to lily toxicity. The conversion of oligo-anuria to polyuria may have been secondary to the administration of furosemide or may have been a natural progression of the underlying disease process and AKI. However, this is the first described survival of oligo-anuric AKI secondary to lily toxicity in a cat without renal replacement therapy. In conclusion, furosemide may be beneficial in the conversion of oligo-anuria to polyuria in AKI secondary to lily toxicity and may prevent the need for renal replacement therapy. Additionally, while the prognosis of anuric AKI secondary to lily toxicity is still poor, a successful resolution may be possible. Further prospective studies are needed to assess the benefits of furosemide on anuric AKI in veterinary medicine.

## Data availability statement

The original contributions presented in the study are included in the article/supplementary material, further inquiries can be directed to the corresponding author.

## Ethics statement

Written informed consent was obtained from the participant/patient(s) for the publication of this case report.

## Author contributions

AT, CD, SS, and RW all contributed to manuscript preparation. All authors contributed to the article and approved the submitted version.

## Footnotes

^1^Calcium gluconate—Fresenius Kabi, Lake Zurich, IL.

^2^Isotonic crystalloid—Dechra Veterinary Products, Overland Park, KS.

^3^Dextrose—International Medication Systems, El Monte, CA.

^4^Cerenia—Zoetis Inc, Kalamazoo, MI.

^5^Albuterol—Glaxosmithkline, Research Triangle Park, NC.

^6^Sodium Bicarbonate—Hospira Inc, Lake Forest, IL.

^7^Unasyn—Meitheal Pharmaceuticals, Chicago, IL.

^8^Humulin R—Lilly USA, Indianapolis, IN.

^9^Pantoprazole—Sun Pharmaceutical Industries Inc, Cranbury, NJ.

^10^Terbutaline—Westward Pharmaceutical Corp, Eatontown, NJ.

^11^Furosemide—Covetrus North America, Dublin, OH.
